# The characterization of the mitochondrial genome of *Calineuria stigmatica* (Plecoptera: Perlidae)

**DOI:** 10.1080/23802359.2019.1659119

**Published:** 2019-09-02

**Authors:** Jinjun Cao, Ying Wang, Guoqiang Zhang, Shanqing Yi, Weihai Li

**Affiliations:** Department of Plant Protection, Henan Institute of Science and Technology, Xinxiang, China

**Keywords:** *Calineuria stigmatica*, mitochondrial genome, characterization

## Abstract

The nearly complete sequence of the mitochondrial DNA of *Calineuria stigmatica* has been completed and annotated in this study. The circular genome is 15,070 bp in length with an A + T content of 61.8% and contains 13 protein-coding genes (PCGs), 22 transfer RNA (tRNA) genes, and two ribosomal RNA (rRNA) genes. The control region can only be assembled partially. All PCGs use normal start codon ATN, while *COI*, *ND1*, and *ND5* use CCG, TTG, and GTG as start codon, respectively. Meanwhile, 10 PCGs use the typical termination codons TAN, except *COII*, *ND4*, *ND5*, which stopped with the incomplete terminaton signal T––. Based on 13 PCGs and two rRNAs using the Bayesian (BI) method supported that *C. stigmatica* was closely grouped with four other Acroneuriinae species. Our results provide basic data for further study of phylogeny in Plecoptera.

*Calineuria* Ricker, 1954, a subgenus of *Acroneyria* Pictet, 1841, was later elevated to genera by Illies (1965). Uchida (1983) transferred the species *A. stigmatica* to the genus *Calineuria*. It is a small genus currently including seven species and is known from the Nearctic, eastern Palearctic, and Oriental regions, and *Calineuria stigmatica* is considered rare in the stonefly groups which only distributed in Japan (Uchida 1983; DeWalt et al. 2019). Mitochondrial DNA is a powerful molecular tool for genetic research and has proven to be useful for rapid identification and genetic source determination (Cameron 2014). To date, nine perlid but only four species from Acroneuriinae have been published previously, and the genus *Calineuria* has not yet been involved (Cao, Li, et al. 2019; Cao, Wang, et al. 2019; Elbrecht et al. 2015; Huang et al. 2015; Li et al. 2019; Qian et al. 2014; Wang, Ding, et al. 2016; Wang, Wang, et al. 2016). In this study, we characterized the mitochondrial genome of *C. stigmatica* by high-throughput sequencing for the first time. This study would supply important basis for conservation genetics of *C. stigmatica*, and promote the phylogenetic analysis of Plecoptera.

The male adult sample of *C. stigmatica* was collected from Yamagata Prefecture (37.951°N, 139.698°E), Japan in 2015 by Xingyue Liu. The thorax muscle of the specimen was used to extract total genomic DNA. Vouchers consisting of the remaining stoneflies (No. Vhl-0064) were deposited in the Entomological Museum of Henan Institute of Science and Technology (HIST), Henan Province, China.

The annotated mitogenome of *C. stigmatica* is 15,070 bp in length, which has been deposited into GenBank with the accession number MG677941. It encoded 37 genes as in other insect mitogenomes, including 13 PCGs, 22 tRNA genes, two rRNA genes, and a partial control region. It had a base composition of A 33.9%, T 27.9%, C 25.2%, and G 13.0%. The A + T content of PCGs, tRNAs, and rRNAs was 60.1, 67.0, and 67.5%, respectively. All PCGs use ATG as start codon except the *ND1* and *ND5* genes used TTG and GTG. Ten PCGs used the typical termination codons TAA or TAG, whereas only three PCGs (*COII*, *ND4*, and *ND5*) stop with the signal T––. The 22 tRNA genes size varied from 64 to 72 bp, comprising a total length of 1475 bp. In addition to the control region, there were 44 nucleotides dispersed in eight intergenic spacers, ranging from 1 to 16. Gene overlaps were also found at sixteen gene junctions involving 44 nucleotides with the longest overlap (eight nucleotides) between *tRNA^Trp^* and *tRNA^Cys^*.

The phylogenetic relationship of *C. stigmatica* was constructed with the sequences of 13 PCGs and two rRNAs from 10 perlid stoneflies (including an unpublished species *Etrocorema hochii*) and two species as outgroups by MEGA 7.0 (Kumar et al. 2016) using the Bayesian (BI) method to conduct the topology tree (Figure 1). Our result showed that *C. stigmatica* was closely grouped with four other Acroneuriinae species, which is in accordance with the traditional morphological classification. More mitogenome information of Perlidae species is needed for the further phylogenetic studies.

**Figure 1.**
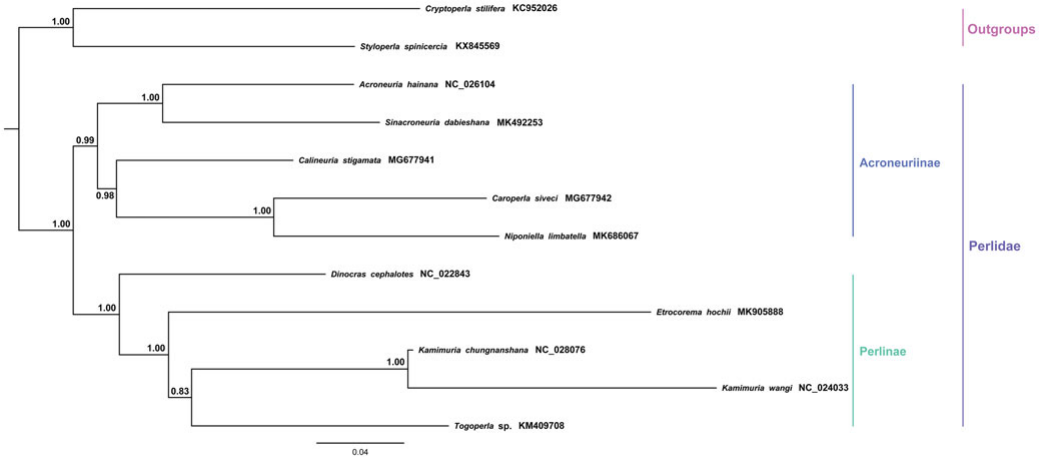
Phylogenetic analyses of *Calineuria stigmatica* based on the concatenated nucleotide sequences of the 13 PCGs and two rRNAs. The NCBI accession number for each species is indicated after the scientific name.
